# Endoscopic Classifications of Early Gastric Cancer: A Literature Review

**DOI:** 10.3390/cancers14010100

**Published:** 2021-12-26

**Authors:** Mary Raina Angeli Fujiyoshi, Haruhiro Inoue, Yusuke Fujiyoshi, Yohei Nishikawa, Akiko Toshimori, Yuto Shimamura, Mayo Tanabe, Haruo Ikeda, Manabu Onimaru

**Affiliations:** Digestive Diseases Center, Showa University Koto Toyosu Hospital, 5-1-38 Toyosu, Koto-ku, Tokyo 135-8577, Japan; haru.inoue@med.showa-u.ac.jp (H.I.); yusukefujiyoshi@yahoo.co.jp (Y.F.); nishikawa6@med.showa-u.ac.jp (Y.N.); akiko.toshimori.2010@gmail.com (A.T.); yutoshimamura1219@gmail.com (Y.S.); mayo.tanabe@gmail.com (M.T.); h.ikeda214@gmail.com (H.I.); mnbonmr0827@gmail.com (M.O.)

**Keywords:** early gastric cancer, endoscopy, classification, diagnosis, endocytoscopy, magnifying endoscopy, narrow band imaging

## Abstract

**Simple Summary:**

Throughout the years, endoscopic technologies have advanced to facilitate better assessment of gastric lesions and early detection of gastric cancer. With improvements in conventional white light endoscopy, we have also witnessed the development of newer endoscopic diagnostic modalities, giving rise to several classifications for early gastric cancer. Different endoscopic classifications of early gastric based on several endoscopic diagnostic modalities were included in this review. In addition to this, newer and novel endoscopic classifications that were specifically developed for the stomach for assessing and diagnosing gastric lesions have also been included. Illustrative representations of each classification have also been provided to aid readers in better understanding of these endoscopic classifications of early gastric cancer.

**Abstract:**

Endoscopic technologies have been continuously advancing throughout the years to facilitate improvement in the detection and diagnosis of gastric lesions. With the development of different endoscopic diagnostic modalities for EGC, several classifications have been advocated for the evaluation of gastric lesions, aiming for an early detection and diagnosis. Sufficient knowledge on the appearance of EGC on white light endoscopy is fundamental for early detection and management. On the other hand, those superficial EGC with subtle morphological changes that are challenging to be detected with white light endoscopy may now be clearly defined by means of image-enhanced endoscopy (IEE). By combining magnifying endoscopy and IEE, irregularities in the surface structures can be evaluated and highlighted, leading to improvements in EGC diagnostic accuracy. The main scope of this review article is to offer a closer look at the different classifications of EGC based on several endoscopic diagnostic modalities, as well as to introduce readers to newer and novel classifications, specifically developed for the stomach, for the assessment and diagnosis of gastric lesions.

## 1. Introduction

Gastric cancer is one of the most commonly diagnosed cancer, ranking fifth worldwide, and the fourth leading cause of cancer-related deaths [1]. Although a decreasing trend has been observed globally, its prevalence remains high in several parts of the world such as Asia, Eastern Europe, and South America [1]. Therefore, optimizing detection and diagnosis of early gastric cancer remains fundamental in improving prognosis and survival outcomes.

Early gastric cancer (EGC) is defined as an invasive gastric cancer that is limited to the gastric mucosal and submucosal layer, irrespective of lymph node metastasis. Definitive diagnosis of EGC is still based on the gold standard histopathological examination [2]; however, endoscopic technologies have been continuously advancing throughout the years to enhance the detection and diagnosis of gastric lesions. Techniques commonly used are white light endoscopy, magnifying endoscopy with narrow-band imaging (NBI), and chromoendoscopy. White light endoscopy is the standard and conventional endoscopic technique. Chromoendoscopy is an endoscopic technique that utilizes staining methods to enhance the characteristics of gastric lesions, differentiating these from the surrounding normal gastric mucosa. Meanwhile, NBI is a virtual and optical chromoendoscopic technique with a similar purpose to conventional chromoendoscopy, which is to enhance the characteristics of gastric lesions. More advanced techniques, such as confocal endomicroscopy and endocytoscopy, although promising, are utilized less. With the development of different endoscopic diagnostic modalities for EGC, several classifications have been advocated for the evaluation of gastric lesions, aiming for an early detection and diagnosis.

Sufficient knowledge on the appearance of EGC on white light endoscopy is fundamental for early detection and management [3]. Each macroscopic type, discussed in the section below, has typical endoscopic findings for the different types of EGC, such as intestinal or diffuse. On the other hand, those superficial EGC with subtle morphological changes that are challenging to be detected with white light endoscopy, such as changes in the surface structures, may now be clearly defined by means of image-enhanced endoscopy (IEE). By combining magnifying endoscopy and IEE, irregularities in the surface structures can be evaluated and highlighted [4], leading to improvements in EGC diagnostic accuracy.

In this literature review, we offer a closer look at the different endoscopic classifications of EGC based on several endoscopic diagnostic modalities, as well as introduce readers to newer and novel classifications, specifically developed for the stomach, for the assessment and diagnosis of gastric lesions.

## 2. White Light Endoscopy and Macroscopic Assessment

Superficial neoplastic lesions of the stomach are usually asymptomatic and are often detected as incidental findings during screening endoscopy. A superficial gastric cancer is defined as carcinomatous lesion extending through the gastric mucosa and the submucosal layer. Identification and detection of these lesions on white light endoscopy, the standard and conventional endoscopic imaging technique, as well as proper assessment of the gross morphological appearance are keys to better management, prognosis, and outcomes.

The original macroscopic classification for EGC was proposed by the Japanese Endoscopy Society in 1962, which has then become the foundation of the current Japanese macroscopic classification [5]. Lesions of the stomach that demonstrate a superficial appearance (including early or advanced carcinoma, adenoma or dysplasia) upon endoscopic examination are classified as Type 0 [6]. Type 0 lesions are further subclassified, as discussed below.

The rest of the current macroscopic classification, shown in Table 1 with its respective description, is as follows: Type 1 (mass), Type 2 (ulcerative), Type 3 (infiltrative ulcerative), Type 4 (diffuse infiltrative), and Type 5 (unclassifiable).

In this section, we will key in on the widely used endoscopic classification for superficial lesions of the gastrointestinal (GI) tract, the Paris classification.

### Paris Classification

In 2002, a general endoscopic classification of superficial lesions in the GI tract was proposed by a group of experts, paving the way for the development of the Paris Classification [7], which has become the standard for endoscopic and macroscopic assessment of GI lesions.

**Figure 1:**
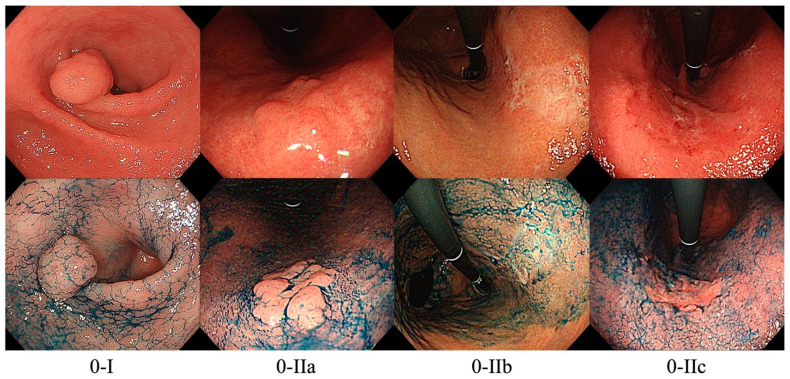
Representative images of the Paris endoscopic classification (upper row: white light only, lower row: same lesions under chromoendoscopy).

As mentioned above, lesions with superficial appearance on endoscopy are classified as Type 0. By including the prefix “Type 0” in the assessment of lesions, endoscopists can distinguish between early and superficial cancer from advanced cancer. Type 0 lesions are further classified into polypoid or non-polypoid lesions. Polypoid lesions, designated as Paris classification 0-I, can either be pedunculated (0-Ip), sessile (0-Is), or semi-pedunculated (0-Isp). 0-I lesions are usually larger in size than benign polyps and exhibits a granular or lobulated shape with a rough surface. 0-I EGC are not very common since polypoid lesions have the lowest risk of progressing into carcinoma; however, 0-I EGCs are difficult to distinguish from hyperplastic polyps. Meanwhile, non-polypoid lesions can be further subdivided into excavated or ulcerative lesions (0-III), or flat lesions (0-II). Flat lesions can either be slightly elevated (0-IIa), at mucosal level (0-IIb), or slightly depressed (0-IIc). Among these, 0-IIb lesions are the most challenging to detect and may be mistaken as atrophic gastritis. Figure 1 shows representative images of the Paris endoscopic classification.

The Paris endoscopic classification has had a growing and significant application in clinical practice since it allows the estimation of invasion depth [7]. Based on previous reports, flat lesions (0-II), especially 0-IIc (slightly depressed), as well as 0-III (excavated or ulcerated) are more likely associated with submucosal invasion [7,8]. To distinguish between 0-IIc and 0-III lesions, endoscopists must analyze the depth of depression or ulceration as well as the surface of the depressed or ulcerated area. In 0-IIc, the depressed area has superficial erosions, involving only the most superficial layers. Meanwhile, in 0-III lesions, there is loss of mucosa and often, the submucosa is also involved [7]. One study by Hu et al. [9] demonstrated that 0-IIc lesions is a risk factor the development of high-grade dysplasia or carcinoma, therefore, endoscopic resection is recommended. Another study by Kim reported that 70–80% of EGCs are 0-IIc lesions [10]. Based on these findings, the Paris classification is useful in screening neoplastic from non-neoplastic lesions, with Type 0-IIc being a more specific indication for endoscopic resection [9], whereas for majority of Type 0-III lesions, surgical resection may be necessary. Another advantage of this classification is that it allows classification of lesions with mixed findings, such a depressed lesion with elevated borders or central elevation (0-IIc + IIa), or an elevated lesion with a central depression (0-IIa + IIc). These mixed type lesions, especially 0-IIa + IIc, generally have a poorer prognosis and a higher risk of a large invasion into the submucosal layer.

Previous studies have shown that the use of the Paris classification during white light endoscopic examination in the detection of EGC has yielded a sensitivity and specificity of 71.2% and 99.1%, respectively [11,12]. Another study showed that the overall accuracy for predicting the invasion depth of EGC based on this classification was 78.0% [13]. These results showed that utilizing the Paris classification during white light endoscopy is an effective screening method with a high precision for the diagnosis of EGC; hence, the reason why it is still the most widely used endoscopic classification for identifying and describing GI lesions.

## 3. Magnifying Endoscopy with NBI

Due to the development of magnifying endoscopy with NBI, diagnostic accuracy for EGC has improved since it allows the identification of subtle morphological changes in the gastric mucosa, prediction of histology and delineation of the lateral spread of the lesion. Since magnifying NBI allows closer evaluation and assessment of the gastric mucosal surface, lesions or mucosal changes that may be missed during white light endoscopy can be detected. Under magnifying endoscopy and NBI, a normal mucosa demonstrates regular arrangement of small, round pits surrounded by collecting venules and a subepithelial capillary network creating a honeycomb appearance [14]. According to Kaise et al. [15], three criteria can be utilized to detect superficial EGC by magnifying NBI, which include: (1) disappearance of fine mucosal structure, (2) microvascular dilation, and (3) heterogenous shape of vessels. These three criteria yielded excellent sensitivity and specificity of 92.9% and 94.7%, respectively [15].

Magnifying endoscopy with NBI has shown superior diagnostic accuracy over the conventional white light endoscopy in several previously reported studies [16], with a sensitivity and specificity of 86% and 96%, respectively. However, combination of conventional white light endoscopy and magnifying endoscopy with NBI yielded a much better and enhanced sensitivity, specificity, and diagnostic accuracy of 95.0%, 96.8% and 96.6%, respectively [13]. While NBI is widely common, full magnifying function (×80) may not be available in a number of facilities. This full magnifying function is sometimes necessary to obtain an even closer and clearer visualization of microvascular and microsurface patterns. It should also be noted that NBI is a feature of endoscopes from Olympus Corporation. Similar methods are available, such as flexible spectral imaging color (FICE) and blue laser imaging (BLI) from Fujifilm, and i-Scan from Pentax; however, there is still insufficient evidence regarding these techniques compared to other endoscopic imaging techniques, and further studies are still warranted.

In this section, we will take a closer look at two previously established classifications of EGC with the use of magnifying endoscopy and NBI, as well as a novel classification.

### 3.1. VS Classification

The Vascular Surface (VS) Classification, proposed in 2009 by Yao et al. [17], has become an established diagnostic classification in characterizing superficial gastric lesions and differentiating them into cancer or non-cancer with the use of magnifying endoscopy. In this classification, the microvascular (MV) pattern and microsurface (MS) pattern are assessed independently to maximize the advantages of magnifying endoscopy and NBI.

Based on VS Classification, typical endoscopic findings of EGC include the presence of a demarcation line, and identification of irregular MV and MS pattern inside the demarcation line. According to Yao et al., 97% of EGC present with these typical endoscopic findings when assessed using this classification [17]. To further supplement this, Nakayoshi et al. reported a more detailed description of the MV pattern, subdividing it into two types: fine network pattern (showing a mesh formation) and corkscrew pattern (showing a tortuous pattern with no connections) [18]. These characteristics have been summarized in Table 2.

Since VS Classification constitutes important and useful characteristics, it facilitates the detection and diagnosis of small cancerous lesions of ≤5 mm in size as well as EGC of 0-IIb macroscopic type, which is challenging to be diagnosed in conventional white light endoscopy [19]. Differentiating intestinal-type cancer from diffuse-type cancer is also possible by assessing the MV pattern (fine network pattern for intestinal-type; corkscrew pattern for diffuse-type). In addition, this classification also allows preoperative assessment and evaluation of the lesion’s borders.

Studies on the diagnostic performance of magnifying NBI using the VS classification have shown satisfactory to excellent accuracy rates, ranging from 79% to more than 95%, with a sensitivity and specificity of 95% and 96%, respectively [13,20,21]. On the other hand, according to a multicenter prospective study, one limitation of the clinical application of VS Classification are flat discolored lesions of the undifferentiated type [21]. Nonetheless, VS Classification remains, without a doubt, the standard method of evaluating gastric lesions by utilizing magnifying NBI.

### 3.2. MESDA-G

**Figure 2:**
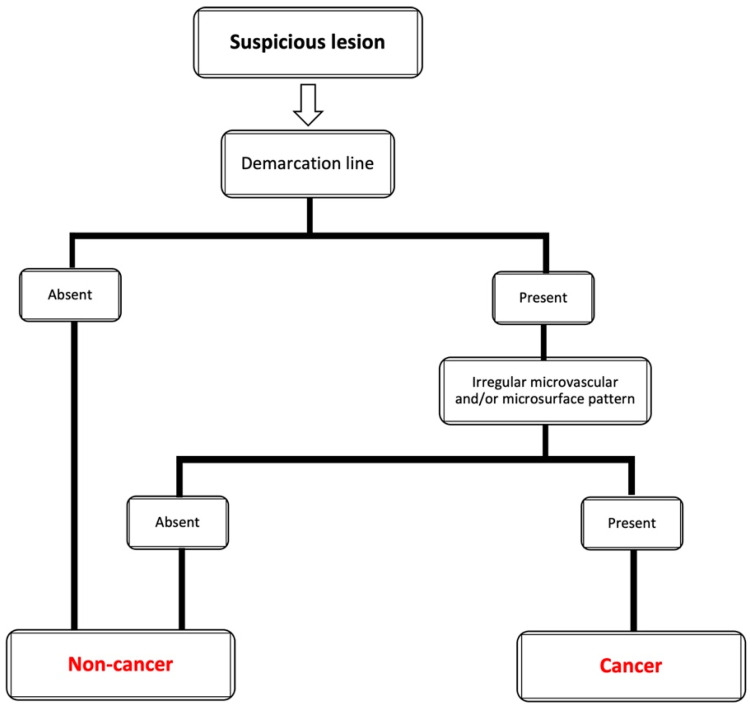
Magnifying endoscopy simple diagnostic algorithm for early gastric cancer (MESDA-G) [22].

With the establishment of the VS Classification, the Japanese Gastric Cancer Association (JGCA), Japan Gastroenterological Endoscopy Society (JGES) and the World Endoscopy Organization (WEO) proposed an evidence-based standardized algorithm for the diagnosis of EGC [3], which was named the Magnifying Endoscopy Simple Diagnostic Algorithm for Early Gastric Cancer (MESDA-G) [22]. This algorithm applies the VS Classification principles in the assessment of a suspicious gastric lesion. Figure 2 shows the MESDA-G algorithm.

According to the MESDA-G, if a suspicious lesion in the stomach is detected, the presence or absence of a demarcation line should be specifically determined through the use of magnifying endoscopy. If a demarcation line is not appreciated, the lesion should be diagnosed as non-cancer. If a clear demarcation line is observed, the MV and MS pattern inside the demarcation line should be assessed independently whether they are regular, irregular, or absent. An irregular MV and/or MS pattern demonstrates that the lesion is a cancer. Meanwhile, when there is the absence of an irregular MV and/or MS pattern, the lesion is non-cancerous.

MESDA-G using the VS Classification has proven to be a simple and useful diagnostic algorithm for the diagnosis of EGC, most especially, superficial (0-II) gastric cancer [23,24]. It has been shown to have high diagnostic accuracy, high positive predictive value, and high negative predictive value of 95%, 79% and 99%, respectively [22]. On the other hand, the rates for diffuse-type EGC were still unclear [22]; hence, it would be valuable to conduct several future studies on the use of MESDA-G in the diagnosis of diffuse-type EGC. Nonetheless, MESDA-G is still a simple algorithm for the diagnosis of EGC.

### 3.3. Unified Magnifying Endoscopic Classification (UMEC)

Recently, our group created and reported a simplified magnifying endoscopic classification which can be used either in esophagus, stomach, or colon, and named it the Unified Magnifying Endoscopic Classification (UMEC) [25]. By basing UMEC on previously established classifications for each respective organ, our group unified the definition of each category across all three organs (esophagus, stomach, colon). Therefore, UMEC is composed of three categories which are the following: non-neoplastic, intramucosal neoplasia, and deep submucosal invasive cancer.

**Figure 3:**
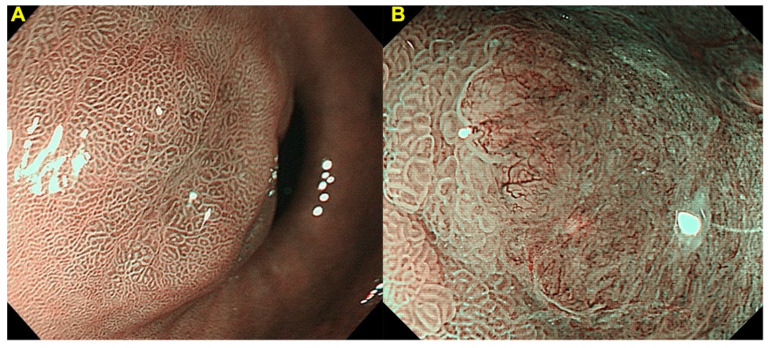
Gastric UMEC: (**A**) UMEC 1/2A is considered as non-cancer and (**B**) UMEC 2B/3 is considered as cancer.

Gastric UMEC, which was based on the VS Classification and MESDA-G, evaluates the absence or presence of a demarcation line, and irregular MV and MS pattern (Figure 3). As shown in Table 3, UMEC 1/2A is identified as non-cancer, and UMEC 2B/3 is identified as cancer. Since there were lack of studies and no sufficient evidence to conclude that non-neoplastic lesions can be distinguished from adenoma by image enhanced magnifying endoscopy, UMEC 1 and 2A were not divided. Similarly, there was no sufficient evidence to conclude that image enhanced magnifying endoscopy is clinically useful in diagnosing invasion depth [26], therefore, UMEC 2B and 3 were not divided.

Our group did a feasibility pilot study [25] on UMEC for the diagnosis of gastric cancer, and our results showed an overall sensitivity, specificity, and accuracy of 90.9%, 89.2%, and 89.5%, respectively. The interobserver agreement was also good (*Kappa* statistic = 0.73, 95%CI: 0.59–0.87). The feasibility pilot study showed that UMEC appears to be a simple classification that can be utilized by non-experts and non-specialized endoscopists. Since UMEC is highly based on MESDA-G, accurate assessment of diffuse-type EGC is still unclear and needs further studies.

To reiterate the purpose of the pilot study, UMEC was not created to replace the current existing organ-specific classifications used by expert and specialized endoscopists. Moreover, the group did not aim to replace conventional histopathological examination (e.g., biopsy) with optical diagnosis using UMEC. Rather, UMEC was created to provide a simpler and more practical classification for non-experts and non-specialized endoscopists who are learning optical diagnosis. Expert and specialized endoscopists can still continue using the existing organ-specific endoscopic classifications. Since this was a feasibility pilot study, future studies on UMEC should be expected to validate the results of our group.

## 4. Endocytoscopy

Endocytoscopy (EC) is one of the more recent advanced novel endoscopic techniques, which offers ultra-high magnification, allowing visualization at the cellular level of the GI mucosa [27]. This technique requires the use of staining methods during the procedure. Based on available literature, EC has been applied in several studies to aid in the diagnosis of gastric lesions. Fasoli et al. reported the use of EC in signet ring cell carcinoma of the stomach [28], which showed absent distinct glandular structure and present peripherally located nucleus surrounded by a cytoplasmic halo. Gastric lymphomas were also assessed with EC by Isomoto et al., showing mucosal aggregation of cellular structures [29]. For gastric intestinal metaplasia, goblet cells are the characteristic EC findings as reported by Chiu et al. [30].

To date, there has still been no consensus on the standardized EC classification for the diagnosis of gastric lesions. In this section, we would like to introduce the readers to the EC classification developed by our group for the diagnosis of EGC by EC.

### EC Classification

**Figure 4:**
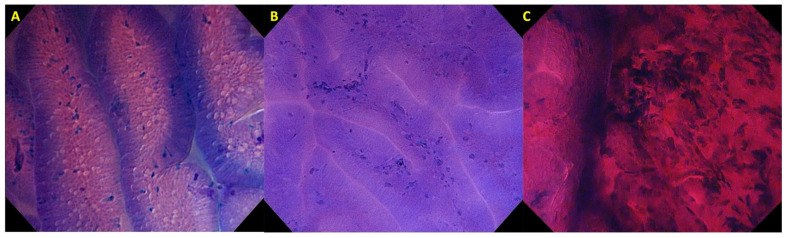
EC Classification: (**A**) EC1 is considered as non-neoplastic, with regularly arranged glands, well-preserved lumen, and poorly stained nuclei; (**B**) EC2 is considered as adenoma, with a more compact glandular arrangement, lumen narrowing and poorly stained nuclei; and (**C**) EC3 is considered as carcinoma with distorted glandular structure and enlarged nuclear sign. The border between the background mucosa and the cancerous area can be noted in this photo.

The first EC classification was developed by Kudo et al. in 2011, for the diagnosis of colorectal lesions [31]. In this classification, structural and cellular atypia, such as lumen morphology and nuclear changes, were assessed to differentiate between non-neoplastic (EC1a and EC1b), and neoplastic (EC2, EC3a, EC3b). By adopting this classification, our group developed a simplified EC classification for the diagnosis of gastric lesions [32,33]. In our simplified classification, we assess the glandular pattern, lumen, and nuclear changes to differentiate between non-neoplastic (EC1), adenoma (EC2), and carcinoma (EC3) (Figure 4). Table 4 shows the characteristics of each gastric EC classification.

Previous studies using the endocytoscopic atypia criteria for the diagnosis of gastric cancer showed favorable results in obtaining in vivo histology, leading to good diagnostic accuracies. One study reported positive and negative predictive values of 100% and 94%, respectively [27]. Another study by Kaise et al. in 2015 showed a diagnostic accuracy rate of 87.3% with a good concordance rate (*Kappa* statistic = 0.682) among four endoscopists [34]. Similarly, our group reported the utilization of EC for the diagnosis of gastric lesions [32]. By basing the diagnosis on our simplified gastric EC classification, our study showed a good diagnostic accuracy (83.7%) and interobserver agreement (*Kappa* statistic = 0.71, 95%CI: 0.50–0.93) for differentiating between gastric cancer and non-malignant cases. These results were compatible to those of conventional histopathology and previous studies, showing that with the use of our simplified gastric EC classification, EC is promising in the diagnosis of EGC.

Although EC appears to be a promising new technique, it is expensive and is only available in a small number of facilities across the world. Obtaining clear images can also be technically challenging due to issues in proper staining method as well as the technical aspect in performing this technique. While training is required to perform EC properly, there are only limited training facilities worldwide. Another major limitation of EC is that the depth of invasion of gastric lesions still cannot be assessed since EC cannot visualize cellular structures beyond the superficial epithelial layer. Acknowledging these areas for improvement, EC deserves further evaluation in future studies.

## 5. Artificial Intelligence

With the continuous development of endoscopic technologies, artificial intelligence (AI) in the field of endoscopy has become a new area of interest. One study by Hirasawa et al., which utilized convolutional neural networks to detect EGC in endoscopic images, showed that the sensitivity of AI for white light endoscopy and magnifying NBI were 92% and 97%, respectively [35]. Meanwhile, a study done by Niikura et al. did not show inferiority nor superiority of AI when compared to expert endoscopists [36]. However, a meta-analysis study showed that the sensitivity and specificity of AI in detecting EGC was 86% and 93%, respectively, concluding that AI was more accurate in detecting EGC compared to expert endoscopists [37]. While majority of studies on AI in the diagnosis of EGC were not real-time, a novel system developed by Wu et al., named ENDOANGEL, has shown to be promising in aiding real-time endoscopic detection and diagnosis of EGC [38].

While AI has further paved the way for the advancement of endoscopic technologies, more studies are warranted to establish definite roles and limitations of AI and endoscopists. Ideally, a balanced and cooperative interaction between AI and endoscopists is desirable to achieve an improved and optimal detection and diagnosis of EGC.

## 6. Conclusions

Throughout the years, endoscopic technologies have advanced to facilitate better assessment of gastric lesions and early detection of gastric cancer. With improvements in conventional white light endoscopy, we have also witnessed the development of newer endoscopic diagnostic modalities, giving rise to several classifications for EGC that were discussed in this review article. From the most known and used Paris classification for white light endoscopy, to the standard magnifying NBI classifications in Japan (VS classification and MESDA-G), and finally, to novel classifications such as UMEC and EC classification; all these classifications have a common denominator, which is to facilitate better detection of EGC. AI in the field of endoscopy has also started developing, potentially providing us an even more improved detection of EGC, although still needing further studies. Nonetheless, adequate knowledge and application to clinical practice of the different classifications of EGC based on the different endoscopic modalities currently available are still fundamental for proper management, improved prognosis, and better survival outcomes.

## Figures and Tables

**Table 1:** Japanese macroscopic classification.

Classification	Description
Type 0 (Superficial)	Superficial lesions involving only the mucosa and the submucosa
Type 1 (Mass)	Polypoid lesions attached to a wide base, with sharp demarcation from surrounding mucosa
Type 2 (Ulcerative)	Ulcerated lesions with raised margins and demarcation line
Type 3 (Infiltrative ulcerative)	Ulcerative infiltrating lesions without clear and definite margins
Type 4 (Diffuse infiltrative)	Nonulcerative diffusely infiltrating lesions without clear and definite margins
Type 5 (Unclassifiable)	Advanced carcinomas that cannot be classified into any of the above types

**Table 2:** VS Classification.

Characteristics	Description
Demarcation line	Present
Microsurface pattern	Irregular
Microvascular pattern	Irregular Fine network pattern (mesh formation) Corkscrew pattern (tortuous pattern with no connections)

**Table 3:** Outline of UMEC in the stomach.

**General UMEC**		**UMEC1**	**UMEC2**		**UMEC3**
			**UMEC2A**	**UMEC2B**	
Expected Histology		Non-neoplastic lesion	Intra mucosal neoplasia		Deep submucosal invasive Cancer
			Benign to low grade neoplasia	High grade neoplasia to Intramucosal cancer (shallow submucosal cancer)	
**Gastric UMEC**		**UMEC 1/2A**		**UMEC 2B/3**	
Endoscopic finding	DL	Absent	Present	Present	
	Vascular and Surface	-	Regular	Irregular microvascular pattern and/or Irregular microsurface pattern	
Expected Histology		Non-cancer		Cancer	

UMEC: Unified Magnifying Endoscopic Classification; DL: demarcation line.

**Table 4:** Gastric EC Classification.

Characteristics	EC1	EC2	EC3
Glandular pattern	Regularly arranged glands with consistent pattern	Recognizable glandular structure with more compact arrangement	Distortion and loss of glandular structure
Lumen	Well-preserved lumen	Lumen narrowing (Slit-like)	No recognizable lumen
Nuclei	Uniform pattern of small, round, poorly stained nuclei with homogenous size	Small, round, poorly stained nuclei with pseudostratification	Hyperchromatic, disarranged nuclei with heterogeneity in size and shape, significant swelling of the nuclei = “enlarged nuclear sign”
Expected Histology	Non-neoplastic	Adenoma	Carcinoma

EC: endocytoscopy.

## Data Availability

Data available in publicly accessible repository. The data presented in this study are openly available in PubMed.
